# Over-expression of poplar *NAC15* gene enhances wood formation in transgenic tobacco

**DOI:** 10.1186/s12870-019-2191-2

**Published:** 2020-01-08

**Authors:** Wenjing Yao, Dawei Zhang, Boru Zhou, Jianping Wang, Renhua Li, Tingbo Jiang

**Affiliations:** 1 State Key Laboratory of Tree Genetics and Breeding, Northeast Forestry University, 51 Hexing Road, Harbin, 150040 China; 2 Co-Innovation Center for Sustainable Forestry in Southern China/Bamboo Research Institute, Nanjing Forestry University, 159 Longpan Road, Nanjing, 210037 China; 3 Department of Agronomy, University of Florida, 2033 Mowry Road, Gainesville, FL 32610 USA

**Keywords:** *Populus simonii×P. nigra*, NAC, Transcription factor, Lignin, Wood formation

## Abstract

**Background:**

NAC (NAM/ATAF/CUC) is one of the largest plant-specific transcription factor (TF) families known to play significant roles in wood formation. Acting as master gene regulators, a few NAC genes can activate secondary wall biosynthesis during wood formation in woody plants.

**Results:**

In the present study, firstly, we screened 110 differentially expressed NAC genes in the leaves, stems, and roots of di-haploid *Populus simonii×P. nigra* by RNA-Seq. Then we identified a nucleus-targeted gene, *NAC15* gene, which was one of the highly expressed genes in the stem among 110 NAC family members. Thirdly, we conducted expression pattern analysis of *NAC15* gene, and observed *NAC15* gene was most highly expressed in the xylem by RT-qPCR. Moreover, we transferred *NAC15* gene into tobacco and obtained 12 transgenic lines overexpressing *NAC15* gene (TLs). And the relative higher content of hemicellulose, cellulose and lignin was observed in the TLs compared to the control lines containing empty vector (CLs). It also showed darker staining in the culms of the TLs with phloroglucinol staining, compared to the CLs. Furthermore, the relative expression level of a few lignin- and cellulose-related genes was significantly higher in the TLs than that in the CLs.

**Conclusions:**

The overall results indicated that *NAC15* gene is highly expressed in the xylem of poplar and may be a potential candidate gene playing an important role in wood formation in transgenic tobacco.

## Background

As one of the most widely and environmentally natural materials, wood is generally used in the construction, paper-making, transportation, chemical industry, military, aerospace and other industries, as well as the production of various wood products, such as agricultural tools, furniture, handicrafts, and musical instruments. Woody biomass can also be utilized as a sustainable and carbon-neutral resource for bioenergy [1]. The demand for wood always increases as it is a cost-effective and renewable resource for industry and energy [2]. There are mainly two kinds of cells with secondary cell walls in the process of wood formation, fibres and tracheary elements. The formation of the two types of cells goes through cell expansion, deposition of secondary walls, lignification and programmed cell death (PCD) [3]. Understanding the process of wood formation contributes to wood property and production, which has significant implication in tree biology and biotechnology.

As a model tree, *Populus* is usually used to understand the unique processes that occur in woody plants, including wood formation [4, 5]. The molecular and genetic mechanisms regulating wood formation in *Populus* have been studied by developmental genetic, genomic and biochemical approaches [6]. The identification of expressed sequence tags (ESTs), hormones and genes regulating wood formation is getting popularity in *Populus* [7–9]. For example, 4% of 5692 ESTs from two poplars were identified to be involved in various processes of cell wall formation, such as lignin and cellulose synthesis [7]. A unique tissue-specific transcript analysis revealed that lignin and cellulose biosynthetic-related genes, transcription factors (TFs) and other potential regulators of xylogenesis were under strict developmental stage-specific transcriptional regulation in poplar [10]. In particular, several TFs such as AUXIN RESPONSE FACTOR (ARF), CLASS III HOMEODOMAIN–LEUCINE ZIPPER (HD-ZIPIII), KANADI (KAN), MYB and NAC might govern the complex networks of transcriptional regulation in wood formation in poplar [9, 11].

NAC family is one of the largest plant-specific TF families known to play significant roles in wood formation [12]. A few NAC genes can activate secondary wall biosynthesis during wood formation acting as master gene regulators, such as vascular-related NAC-domain genes (*VND*) and secondary wall-associated NAC domain genes (*SND*) [13, 14]. Transcriptional profiling indicated there were seven *VND* genes expressed preferentially in the developing vascular tissues in *Arabidopsis* [15]. Out of them, *VND6* and *VND7* are key regulators of xylem vessel differentiation. They regulate the expression of a broad range of genes involved in xylem vessel formation [16, 17]. Two NAC domain TFs, *SND1* and *NST1* (NAC secondary wall thickening promoting factor 1) were proved to function redundantly in regulation of secondary wall synthesis in *Arabidopsis* [18]. Except NAC genes from *Arabidopsis*, many wood-associated NAC domain (*WND*) genes from *Populus* were identified to be master regulators in wood formation. For example, overexpression of two NAC genes from *Populus trichocarpa*, *PtrWND2B* and *PtrWND6B*, leaded to ectopic deposition of cellulose, xylan, and lignin in *Arabidopsis* by inducing the expression of secondary wall-associated TFs and secondary wall biosynthetic genes [14]. Chimeric repressor of a secondary wall-associated NAC gene from *Populus* (*PtSND2*) severely affected wood formation in transgenic *P. davidiana* × *P. bolleana* by down-regulating a number of wood-associated genes [19].

NAC transcription regulators in wood formation precisely coordinate the expression of secondary wall-related genes, which requires fine temporal and spatial regulation [14, 20]. There were 289 putative NAC genes in *Populus trichocarpa*, and most of them showed different temporal and spatial expression patterns [21, 22]. In this study, firstly, we screened differentially expressed NAC genes in the leaves, stems, and roots of di-haploid *Populus simonii×P. nigra* by RNA-Seq. Then we conducted expression pattern analysis of *NAC15* gene in the different tissues by RT-qPCR. Thirdly, we confirmed subcellular localization of *NAC15* gene by particle bombardment. Moreover, we transformed the gene into tobacco through Agrobacterium-mediated method and performed physiological, histological and molecular analysis of transgenic tobacco lines overexpressing *NAC15* gene. The study indicated *NAC15* gene from poplar plays an important role in wood formation in transgenic tobacco.

## Results

### Transcriptome analysis of NAC family in *Populus simonii×P. nigra*

**Fig. 1:**
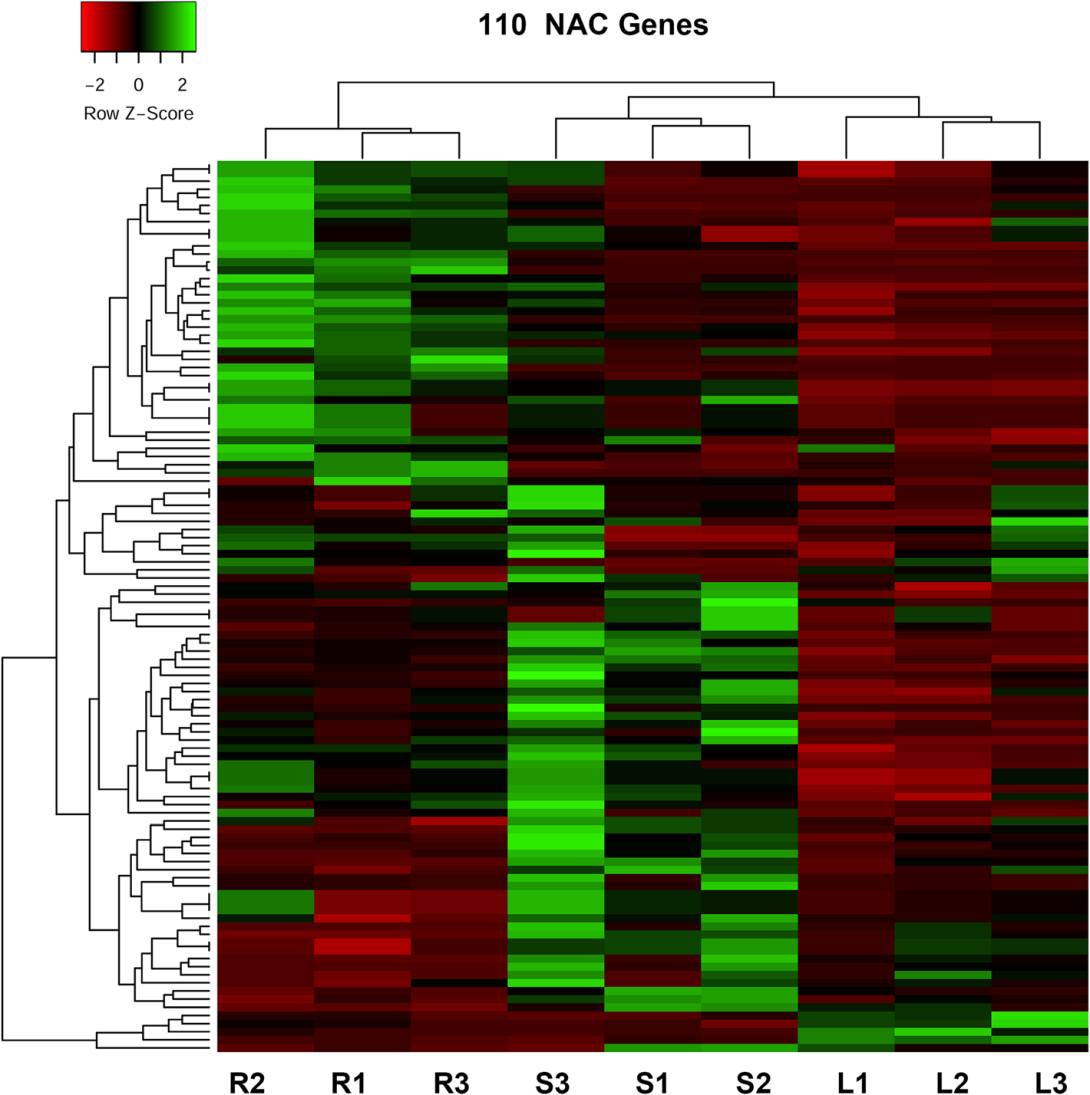
The heatmap of differentially expressed 110 NAC genes in the three tissues of *Populus simonii× P.nigra.* The heatmap was drawn by Heatmapper (http://www.heatmapper.ca/expression/). Red and green colors indicate low and high expression, respectively. R1–3, S1–3 and L1–3 indicate roots, stems and leaves with three biological repeats, respectively

The mRNA abundance of each gene in each sample was profiled as fragment per kilo bases per million reads (FPKM). The FPKM information of all 289 NAC members in the roots, stems and leaves of *Populus simonii×P. nigra* was retrieved from RNA-seq data (Additional file 3: Excel S1). There were a total of 231 NAC genes detected by RNA-Seq. Based on FPKM ≥4 in at least one tissue, 126 out of the 231 genes were screened to count the expression of NAC genes. Out of the 126 genes, there were 115, 123, 118 differentially expressed genes in the comparison pairs between leaves and stems, roots and stems, leaves and roots, respectively. As many as 110 NAC genes were differentially expressed in the three tissues. The heatmap of the 110 genes showed the expression pattern in the leaves and stems can be clustered together, which indicated the genes have similar expression pattern in the two tissues (Fig. 1).

### Phylogenetic analysis of NAC15

**Fig. 2:**
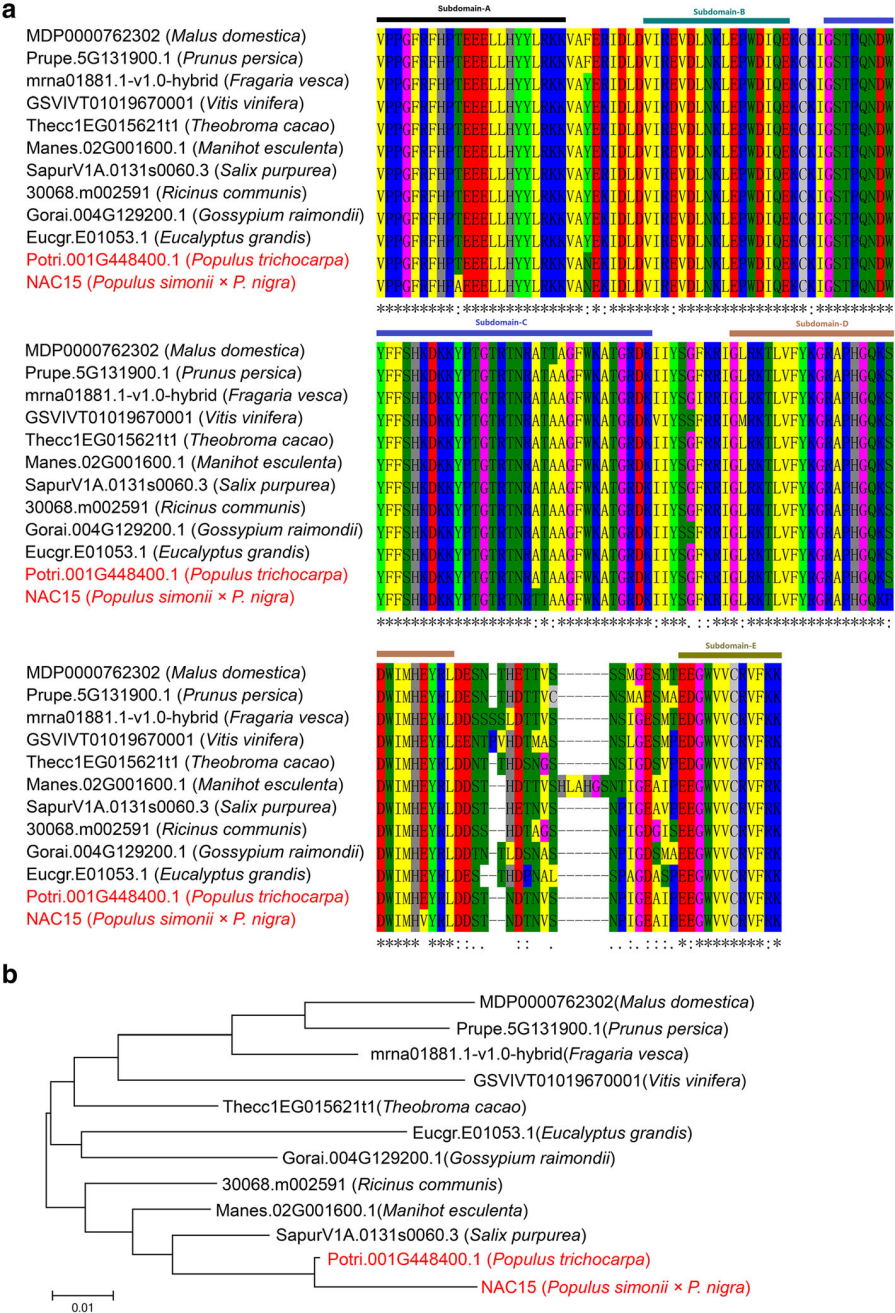
Conserved domain alignment and phylogenetic analysis of NACs from 12 different plant species. The conserved NAC domain can be divided to 5 sub-domains (A-E). The colorful horizontal bars represent the start and end positions of each sub-domain. **a** Domain alignment of NACs by Clustal W; **b** Phylogenetic tree of NACs constructed by Neighbor-Joining method with MEGA 6 program

The 1257 bp coding sequence of *NAC15* gene from *Populus simonii × P. nigra* (Potri.001G448400.1) contains an ORF encoding 418 amino acids. Amino acids sequence blasts indicated that NAC15 from poplar shared 86, 77, 75, 77, 72, 66, 66, 66, 65, 65% sequence similarity with *Salix purpurea* (SapurV1A.0131 s0060.3), *Ricinus communis* (30,068.m002591), *Manihot esculenta* (Manes.02G001600.1), *Theobroma cacao* (Thecc1EG015621t1), *Gossypium raimondii* (Gorai.004G129200.1), *Prunus persica* (Prupe.5G131900.1), *Malus domestica* (MDP0000762302), *Fragaria vesca* (mrna01881.1-v1.0-hybrid), *Eucalyptus grandis* (Eucgr.E01053.1), and *Vitis vinifera* (GSVIVT01019670001), respectively. Multiple amino acids alignment showed that above proteins shared a highly conserved domain of 160 amino acids, namely NAC domains, which can be divided into A-E sub-domains (Fig. 2a). The phylogenetic tree with the top 10 identical protein sequences indicated that NAC15 from poplar had relatively high homology with the proteins from willow, cassava and castor-oil plant, while had relatively low homology with those from wild-strawberry, peach tree and apple tree (Fig. 2b).

### Localization of NAC15 protein

**Fig. 3:**
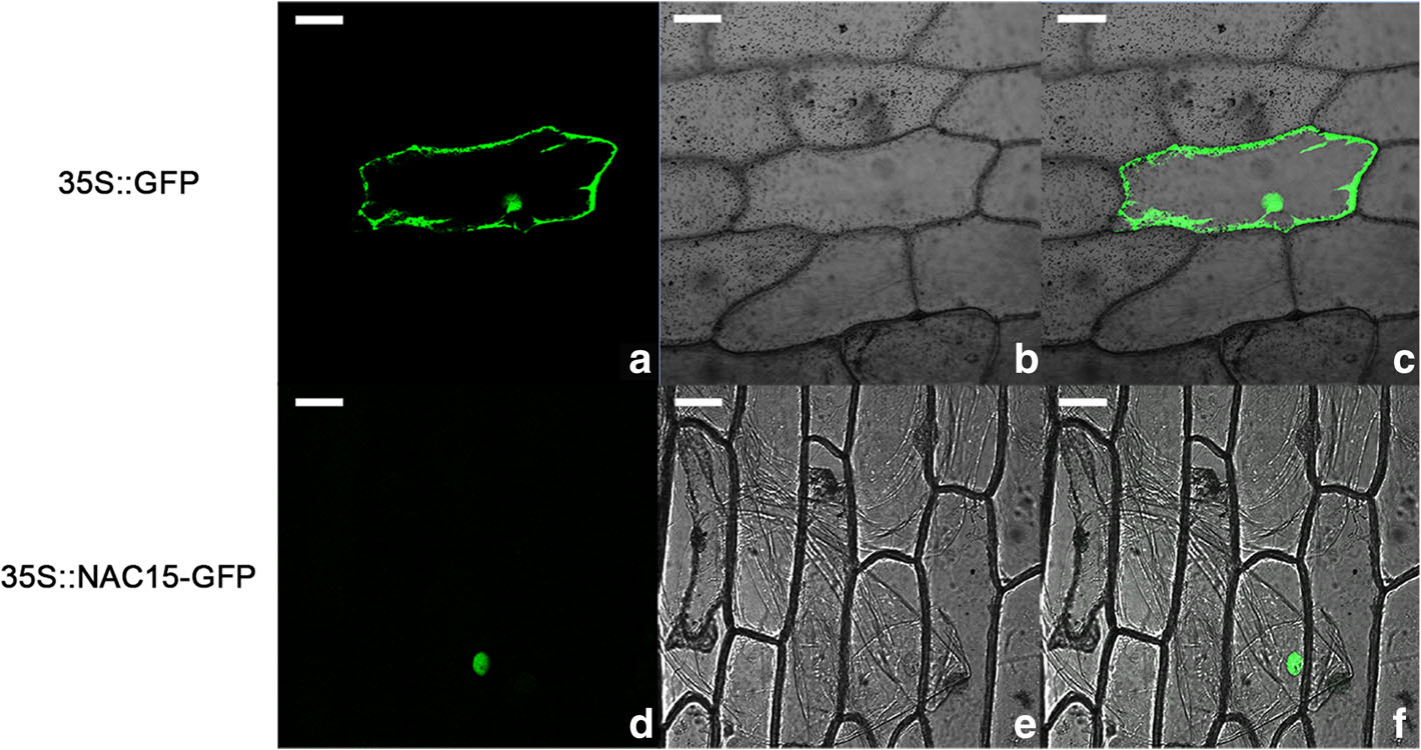
Subcellular localization of NAC15 in onion epidermal cells by particle bombardment. NAC15 was localized to the nucleus. **a-c** The GFP fluorescence signals of 35S::GFP vector; **d-f** The GFP fluorescence signals of 35 s::NAC15-GFP fusion construct. **a** and **d**, dark field; **b** and **e**, bright field; **c** and **f**, overlay of dark field and bright field. Scale bar = 20 μm

As shown in Fig. 3, the fluorescence signal of NAC15-GFP (green fluorescent protein) fusion was detected in the nucleus while the control was fully expressed in the cell, which revealed NAC15 protein was localized to the nucleus. To confirm the result, the NAC15-GFP-transfected onion cells were stained with DAPI and observed under immunofluorescence microscope. The combined fluorescence signal of DAPI and GFP was consistently in the nuclei (Additional file 4: Figure S1), which exactly proved nuclear localization of NAC15.

### Expression pattern analysis of *NAC15* gene

**Fig. 4:**
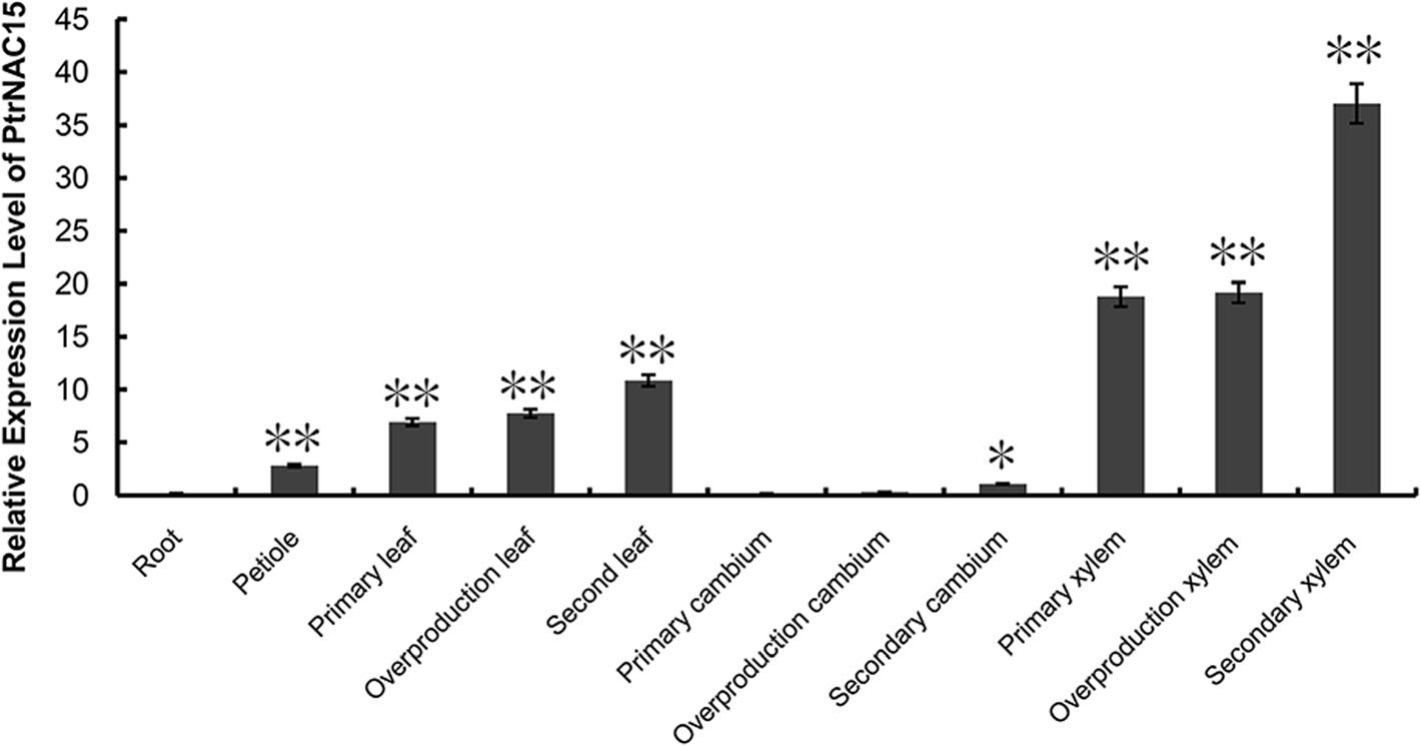
Expression pattern analysis of *NAC15* gene. *NAC15* gene was most highly expressed in the xylem. Mean values and standard errors were calculated from three technical replicates by 2^-△△Ct^ method with three independent biological replicates. * indicates *P* < 0.05, ** indicates *P* < 0.01

*NAC15* gene was differentially expressed in the leaves, stems and roots, and its mRNA abundance was the highest in the stems, followed by leaves and roots based on RNA-Seq. The relative expression level of *NAC15* gene in different tissues at different developmental stage was quantified by RT-qPCR. The results indicated the expression pattern of *NAC15* gene was hugely diverse at different tissues and displayed a rapid decrease from xylems and leaves to cambiums and roots. The highest expression level appeared in the secondary xylems and the lowest was in the roots, which was in accordance with RNA-Seq results in trend. And the highest expression level was about 173 times higher than the lowest (Fig. 4). The relative expression level of *NAC15* gene was also significantly different during developing stages. For example, it was higher in the secondary xylems than that in the primary and crude xylems of poplar (Fig. 4). In conclusion, the expression of *NAC15* gene had spatio-temporal specificity, and its expression pattern may play a pivotal role in the temporal and spatial regulation of wood-associated genes in the process of wood formation.

### Generation of trangenic tobacco overexpressing *NAC15* gene

**Fig. 5:**
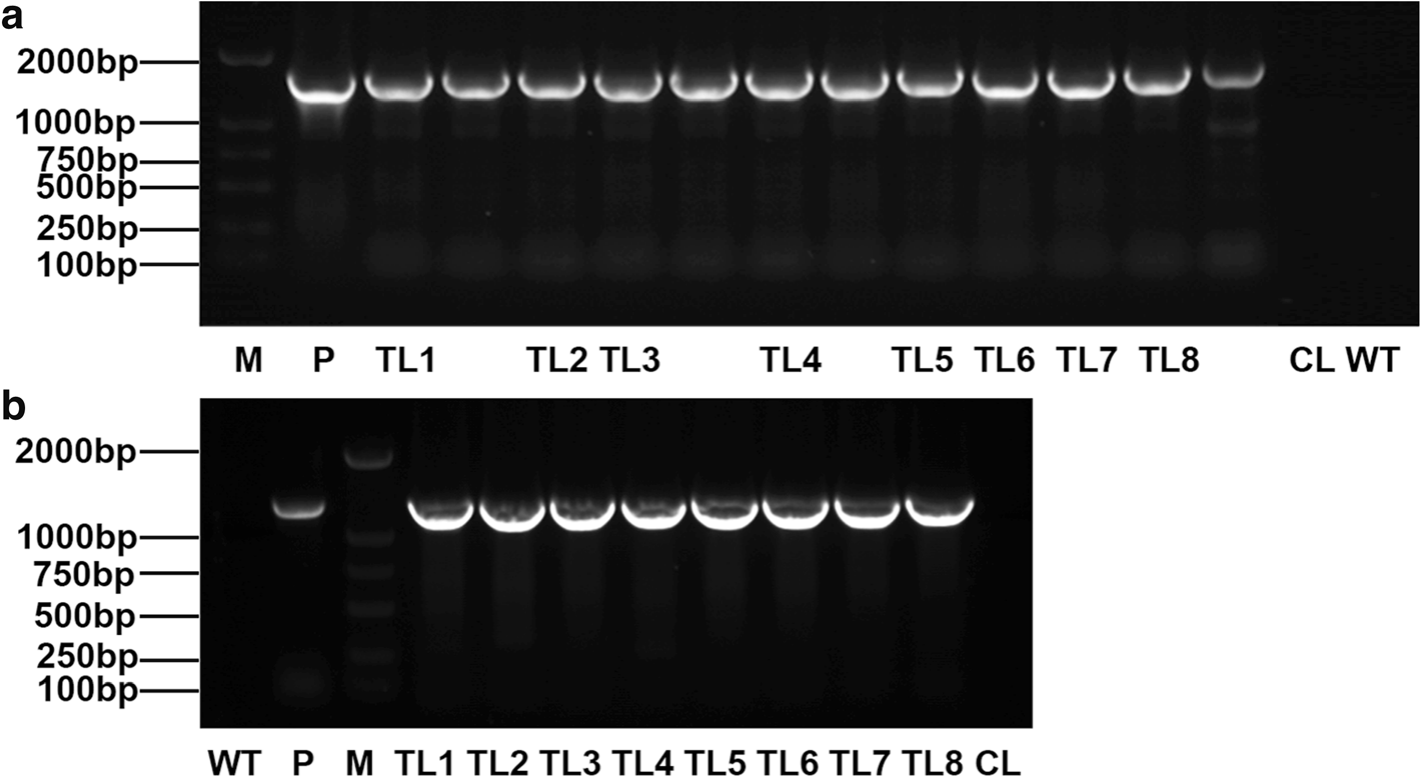
PCR identification of transgenic tobacco lines. **a** PCR detection of the transgenic lines with DNA as template; **b** RT-PCR detection of the transgenic lines with cDNA as template. M, DL2000 marker; P, positive control use pBI121-NAC15 vector as template; TL1–8, transgenic lines; CL, control line; WT, wild type

We obtained 18 transgenic tobacco lines including 12 TLs and 6 CLs. The transgenic tobacco was confirmed by PCR and RT-PCR. As shown in Fig. 5, the expected bands were amplified in the TLs, but not in the CLs and wild type (WT) plants, which proved successful integration of *NAC15* gene in tobacco.

### Gene expression analysis of lignin- and cellulose-related genes by RT-qPCR

**Fig. 6:**
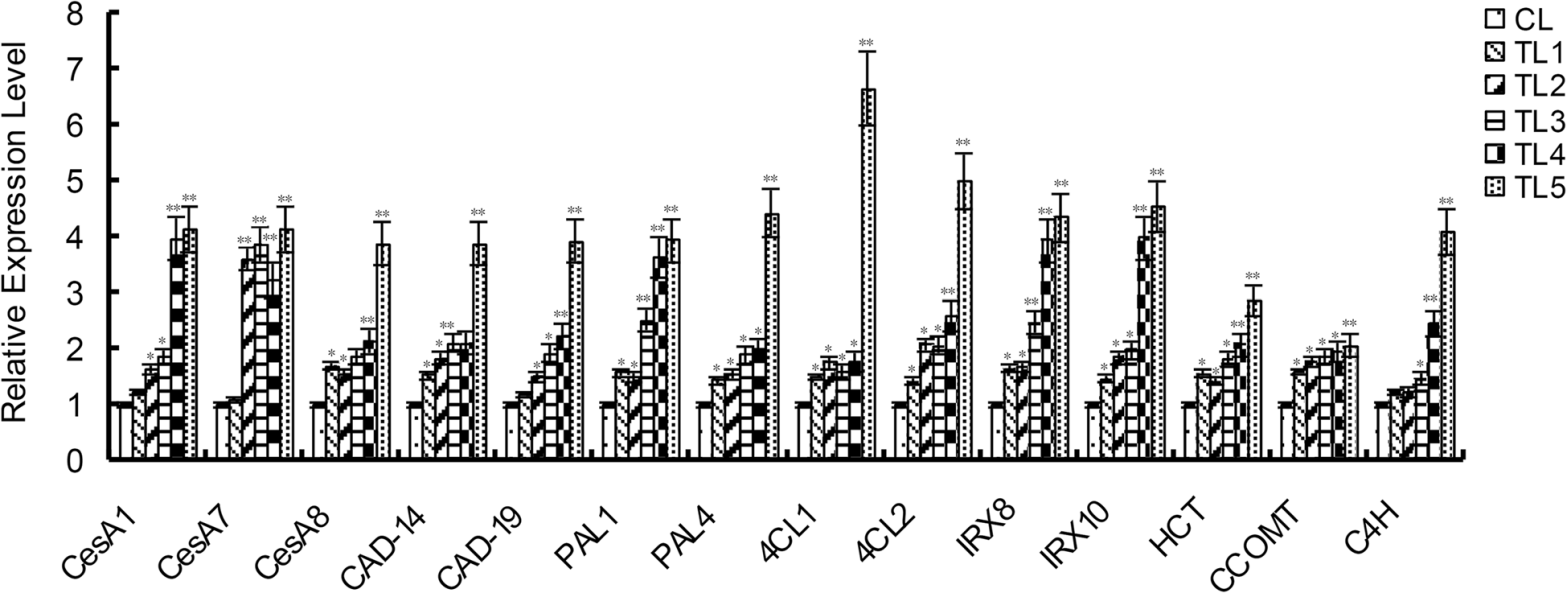
Relative expression level of lignin- and cellulose-related genes. The relative expression level of lignin- and cellulose-related genes was higher in the TLs than that in the CLs. TL1–5, transgenic lines; CL, control line. Mean values and standard errors were calculated from three independent biological experiments. * indicates *P* < 0.05, ** indicates *P* < 0.01

A few lignin- and cellulose-related genes, such as *CesA* (Cellulose synthase), *C4H* (Cinnamate 4-hydroxylase), *CAD* (Cinnamyl alcohol dehydrogenase), *PAL* (Phe ammonia-lyase), *CL* (Coumarate: coenzyme A ligase), *CCOMT* (Caffeoyl-CoA O-methyltransferase) etc. (Additional file 1: Table S1) were required for secondary wall biosynthesis in plants [23, 24]. Taken *CesA* as an example, *PtoCesA3* was highly expressed during primary cell wall formation and was proved to be associated with growth and wood properties of *Populus tomentosa* [25]. *PAL1* and *PAL2* were identified to have relationship with tissue-specific lignin synthesis [26]. RT-qPCR was conducted to detect the relative expression level of lignin- and cellulose-related genes in the transgenic plants. The results indicated the relative expression level of *CesA*, *CAD*, *PAL*, 4*CL*, and *C4H* etc. in the TLs was significantly higher than that in the CLs (Fig. 6).

### Histological analysis of transgenic tobacco overexpressing *NAC15* gene

**Fig. 7:**
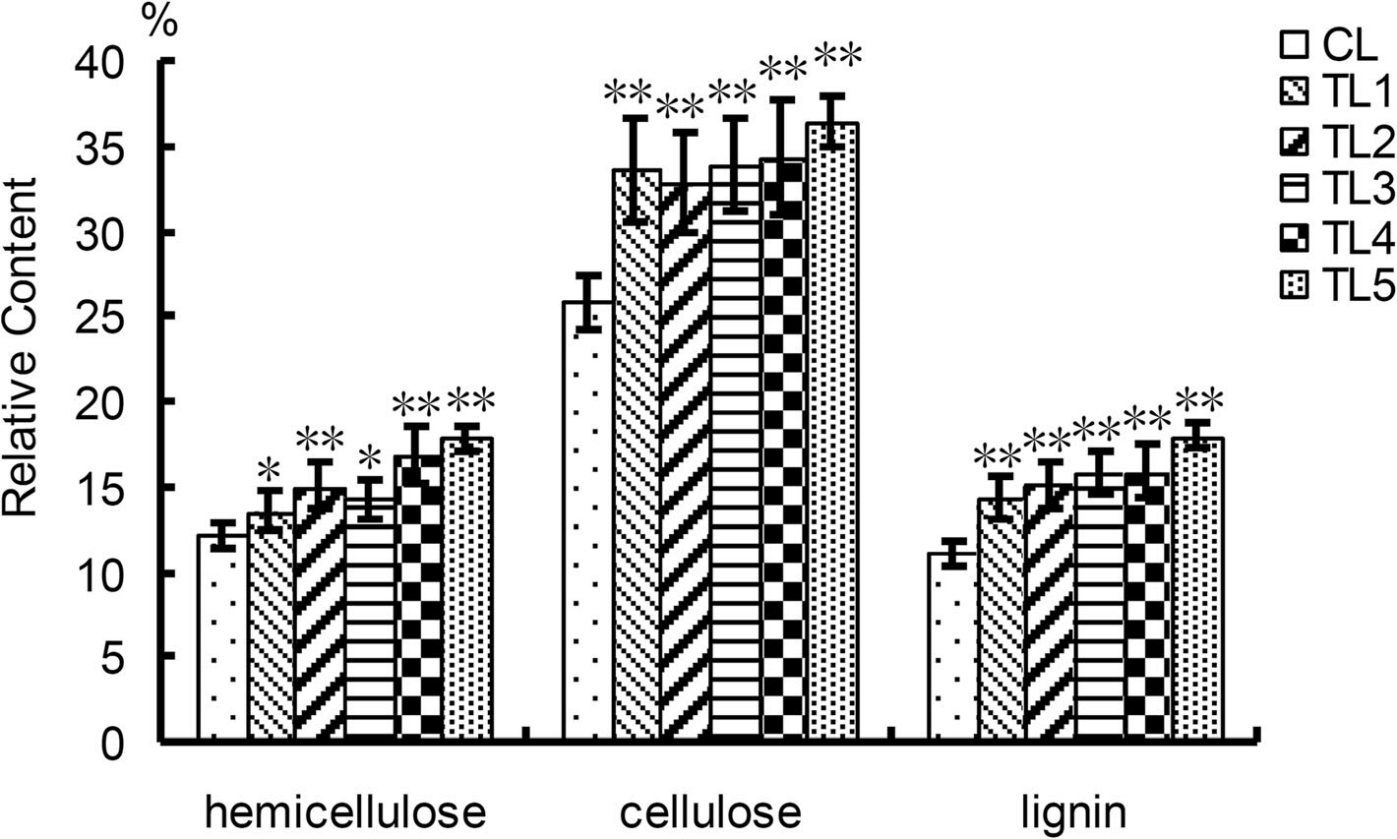
Wood property comparison of tobacco plants. The relative content of hemicellulose, cellulose and lignin was higher in the TLs than that in the CLs. TL1–5, transgenic lines; CL, control line. Mean values and standard errors were calculated from three independent biological experiments. * indicates *P* < 0.05, ** indicates *P* < 0.01

There are three types of polymers (hemicelluloses, cellulose and lignin) in the secondary cell wall of plants [23, 27]. Cellulose is the most abundant polysaccharide in plants and its microfibrils can form a main load-bearing network. Hemicellulose mainly consists of xylans, glucans, and mannans. Lignin affects ‘waterproofing’ capacity, mechanical strength, rigidity and environmental protection of plants [23, 27]. The relative content of hemicellulose, cellulose and lignin was determined to compare wood properties between TLs and CLs. The results showed that the relative content of hemicellulose, cellulose and lignin in the TLs was 1.09–1.38, 1.29–1.40, 1.31–1.58 times higher than that in the CLs, respectively (Fig. 7).

### Phloroglucinol-HCl staining

**Fig. 8:**
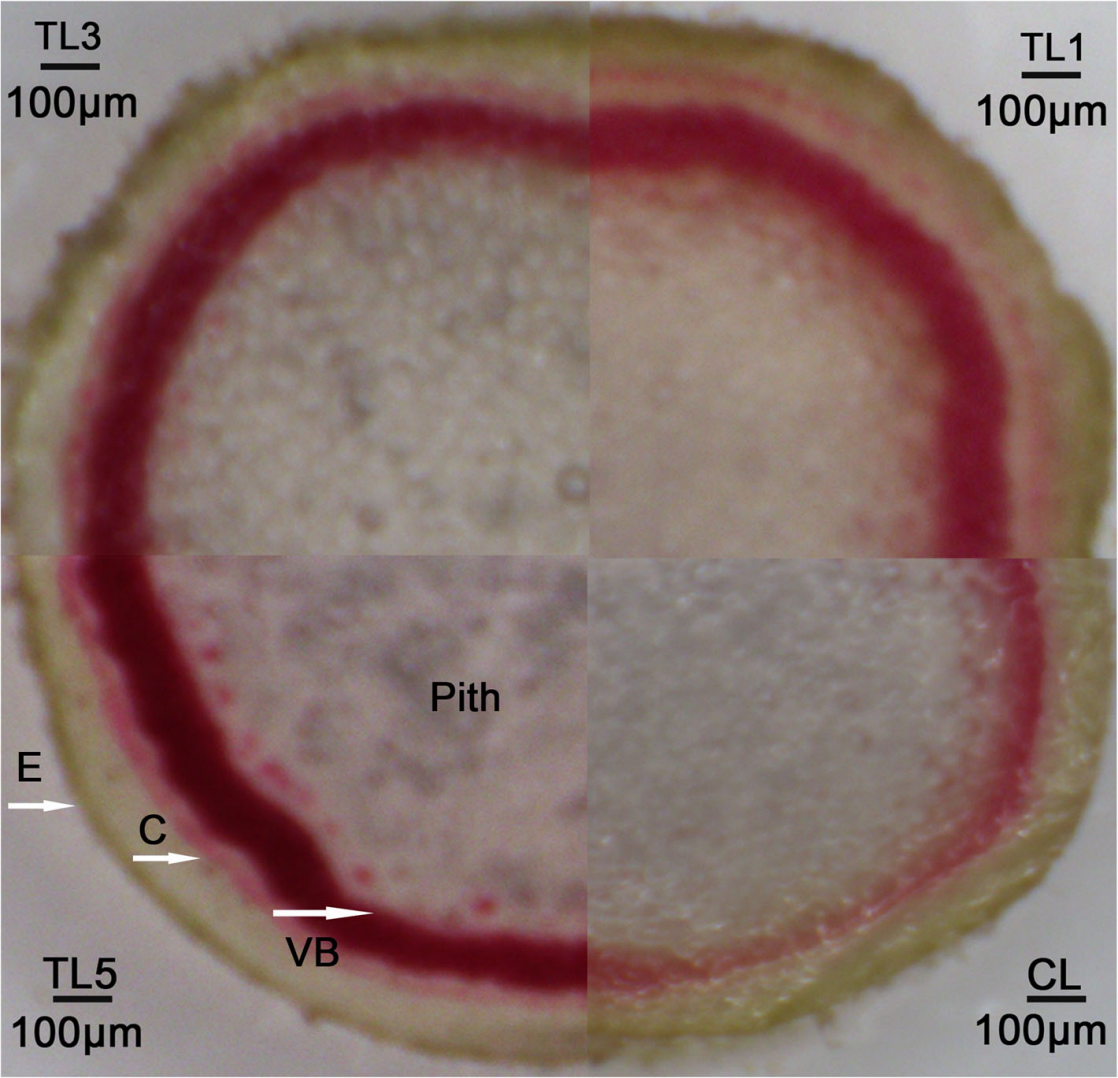
Phloroglucinol staining of tobacco plants. The staining color was obviously deeper in the TLs than that in the CL. And the three TLs showed three levels of staining color, which indicates three levels of lignification. TL1, 3, 5, transgenic lines; CL, control line; E, epidermis; C, cortex; VB, vascular bundles

Phloroglucinol-HCl staining method is commonly used for the characterization of plant lignifications [28]. Therefore, phloroglucinol-HCl staining was conducted to compare wood properties between TLs and CLs in the study. The result showed there was darker staining in the vascular bundles of TLs, compared to the CLs (Fig. 8). It showed three levels of stem lignifications based on the staining color in the TL1, TL3 and TL5, which was in accordance with the relative content of lignin and relative expression level of lignin-related genes.

## Discussion

NAC genes are important plant-specific TFs, which regulate multiple biological processes such as plant developmental process, metabolism process, abiotic stress and defense response [22, 29]. The function of NAC genes in wood formation has come under the spotlight. NAC genes are expressed preferentially in developing wood and differentiating tracheary elements [11, 15]. In particular, *VND*/*NST*/*SND* subfamilies of NAC domain proteins participate in transcriptional control of secondary cell wall formation as master switch [11, 12, 30] (Additional file 5: Figure S2). *VND* proteins control the expression of genes involved in both secondary wall formation and PCD while *NST* genes play pivotal roles in transcriptional regulation of secondary wall formation [11, 16, 31] (Additional file 5: Figure S2). *VND* and *NST* genes with their downstream genes including other NAC domain proteins, MYB proteins, and homeobox proteins form a transcriptional network regulating secondary wall formation during wood formation [9, 30, 31] (Additional file 5: Figure S2).

Considerable effort has been made to shed light on the NAC genes in the wood formation of woody plants. It was proposed the existed reciprocal cross-regulation of *VND* and *SND* multi-gene TF families maintain homeostasis in xylem differentiation in *Populus trichocarpa* [32]. Prominently, wood-associated NAC master switches from poplar (*PtrWNDs*) are preferentially expressed in the developing wood and key regulators of the biosynthesis of cellulose, xylan, and lignin [14] (Additional file 5: Figure S2). Overexpression of *PtrWNDs* led to ectopic deposition of wood components in transgenic poplar [1, 20]. Additionally, dominant repression of *PtrWNDs* caused a drastic reduction of secondary wall thickening in transgenic poplar [13, 20]. *PtrWNDs* can regulate a suite of downstream wood-associated TFs and wood biosynthetic genes to activate entire secondary wall biosynthetic program in *Populus trichocarpa* [14, 20] (Additional file 5: Figure S2). In the study, we identified a nucleus-targeted gene from *Populus simonii×P. nigra*, *NAC15* gene. It was one of the highly expressed NACs in the stem based on RNA-Seq. And expression pattern analysis indicated *NAC15* gene was most highly expressed in the xylem. The relative content of hemicellulose, cellulose and lignin was higher in the TLs than that in the CLs. Phloroglucinol staining showed darker staining in the phloem and xylem of the TLs, compared to the CLs. And the relative expression level of a few lignin- and cellulose-related genes was significantly higher in the TLs than that in the CLs. All the results indicated *NAC15* gene acting as a member of *PtrWNDs* plays a significant role in wood formation in transgenic tobacco.

It was well known that many genes with high protein sequence similarity can be clustered into same sub-group and generally possess similar function. Based on phylogenetic analysis of well-known *Arabidopsis* NAC TFs regulating differentiation of xylem vessels and fiber cells, 16 poplar NAC domain homologs were isolated from *Populus trichocarpa*. Among them, 12 were identified to be *PtrWND* genes [1]. *NAC15* gene was classed into subgroup (V) with *SND1* gene from *Arabidopsis*, which has been demonstrated as a key transcriptional switch regulating secondary wall synthesis in fibers [33, 34]. This subgroup also contains other wood-associated genes, such as *NST1* gene and *NST2* gene, which regulate secondary wall thickenings in *Arabidopsis* [35]. In addition, *NAC15* has high homology with *WND1A* gene, which was identified to regulate cell wall thickening during fiber development in *Populus* species [14, 36]. All above also indicated *NAC15* gene is associated with wood formation in plants.

## Conclusions

Among 289 NAC family members from *Populus simonii × P. nigra*, a total of 115, 123, 118 genes were differentially expressed in the comparison pairs between leaves and stems, roots and stems, leaves and roots, respectively. As many as 110 NAC genes were identified to be differentially expressed in the three tissues. Out of them, *NAC15* gene was highly expressed in the stem. And the gene was confirmed to be nucleus-targeted. The TLs displayed higher content of hemicellulose, cellulose and lignin, compared to the CLs. Phloroglucinol staining also showed an increase of lignification in the vascular bundles of the TLs, compared to the CLs. The relative expression level of a few lignin- and cellulose-related genes such as *CesA*, *CAD*, *PAL*, 4*CL*, and *C4H* etc. was significantly higher in the TLs than that in the CLs. All the results indicated *NAC15* gene from poplar plays an important role in wood formation in transgenic tobacco.

## Methods

### Plant materials and culture

*Populus simonii×P. nigra* is a specific hybrid poplar widely grown in the northeast, northwest and southwest of China. The growing twigs of wild-type *Populus simonii×P. nigra* from one clone of experimental forest of Northeast Forestry University were hydroponic cultured at room temperature with 16/8-h light/dark cycles and 70% relative humidity for two months. The new roots, stems and leaves from the twigs were frozen in liquid nitrogen for RNA-Seq. And the roots, petioles, leaves, xylem and cambiums were harvested for expression pattern analysis. Three biological replicates were prepared for each tissue.

The seeds of wild-type *Nicotiana tabacum* were originated from state key laboratory of tree genetics and breeding of Northeast Forestry University. To prepare sterilized tobacco explants*,* the tobacco seeds were sterilized using 70% (v/v) ethanol for 30 s, followed by NaClO solution (1% NaClO, 0.05% TWEEN20) for 10 min and rinsed using sterile water for 5 times. Then the seeds were placed on 1/2 MS solid medium (pH 5.8–6.0) at 24 ± 2 °C, 16/8-h light/dark cycles for germination. And the germinated seeds were transferred into tissue culture bottles containing 1/2 MS solid medium. The one month old disease free seedlings were used for gene transformation [37].

### NAC expression analysis by RNA-Seq

A total of nine samples including leaves, stems, and roots with respective three biological replicates were shipped with dry ice to GENEWIZ Company (www.genewiz.com) for RNA isolation, mRNA-purification, and RNA-Seq with Illumina Hi-seq platform. The raw sequences were cleaned using Trimmomatic v0.30 [38]. The cleaned reads were aligned to *Populus trichocarpa* reference genome using STAR 2.4.2a [39]. The mRNA abundance of each gene in each sample was quantified as FPKM.

The FPKM information of 289 NAC family members was drawn from RNA-Seq data (Additional file 3: Excel S1). The NACs with FPKM≥4 in at least one tissue were applied to count differentially expressed NAC genes in the three tissues. The fold change (FC) in the different tissues was standardized by Log_2_ FPKM ratios [40, 41]. The hierarchical clustering of the differentially expressed NAC genes in the three tissues was conducted by Heatmapper (http://www.heatmapper.ca/expression/).

### RT-qPCR analysis

The total RNA was extracted using Column Plant RNAout Kit (CAT#:71203, Tiandz, Beijing, China) and reverse-transcribed into cDNA using PrimeScript™ RT reagent Kit with gDNA Eraser (RR047A, Takara, Dalian, China). RT-qPCR experiment was performed by ABI7500 fast real-time PCR detection system using SYBR Premix Ex Taq™II (DRR081A, TaKaRa, Dalian, China). The relative expression level of genes was calculated by 2^-△△Ct^ method with three biological replicates [42]. The primer pairs of poplar *NAC15* gene (NAC15–1), reference gene, and lignin- and cellulose-related genes (Additional file 2: Table S2) were designed based on *Populus trichocarpa* v3.1 in Phytozome12 (https://phytozome.jgi.doe.gov/pz/portal.html).

### Phylogenetic analysis of NAC15 protein

Amino acid sequences of NACs from *Populus trichocarpa* and other species were derived from PlantTFDB (http://planttfdb.cbi.pku.edu.cn/). Multiple alignment of conserved NAC domain was performed by Clustal W [43]. Phylogenetic tree of NAC proteins was constructed by Neighbor-Joining method with MEGA 6 program [44].

### Subcellular localization of NAC15

The coding region of *NAC15* gene without stop codon was cloned into pBI121 vector with specific primers (NAC15–2, Additional file 2: Table S2) and expressed with GFP under the control of CaMV35S promoter. The combined vectors 35S::NAC15-GFP and 35S::GFP as control were transferred into onion epidermal cells by particle bombardment, separately. The fluorescence signal of GFP and DAPI was detected by fluorescence microscopy system (LSM 700, Zeiss, Germany).

### Generation of transgenic tobacco overexpressing *NAC15* gene

The 1515 bp transcript sequence of *NAC15* gene was cloned into pBI121 vector under the control of CaMV35S promoter with specific primers (NAC15–3, Additional file 2: Table S2). The recombined vector and empty vector as control were transformed into EHA105 Agrobacterium strain by electroporation, separately. The transformed EHA105 strain was confirmed by PCR and sequencing.

The tobacco transformation was conducted as following: 1) the leaves from disease free plants at one month old were cut into 1 cm × 1 cm discs and soaked in the positively transformed EHA105 liquid medium (OD 0.3–0.5) for 10 min; 2) the leave discs were dried with sterilized filter paper and put on 1/2 MS solid medium for co-culture in dark for two days; 3) the leave discs were transferred on the pre-cultural medium (1/2 MS solid medium containing 0.5 mg/L 6-BA, 0.05 mg/L NAA and 100 mg/L Kan) until callus emerged; 4) healthy callus were transferred on the shooting medium (1/2 MS solid medium containing 0.1 mg/L 6-BA, 0.05 mg/L NAA and 100 mg/L Kan) until shoots grew; 5) the shoots were transferred into the rooting medium (1/2 MS containing 0.2 mg/L IBA and 100 mg/L Kan) until roots generated; 6) the transgenic tobacco seedlings were confirmed by PCR and RT-PCR [45]. The specific primer pairs (NAC15–4) for PCR and RT-PCR were list in the Additional file 2: Table S2.

### Determination of secondary wall composition

The relative content of lignin, hemicellulose and cellulose in tobacco plants was measured at maturation stage with three biological replicates. The sample preparations, determination procedures and calculation formulas referred to the description by Sukjun et al. [46].

### Histological analysis

Histological staining was conducted in the tobacco at growth period with three biological replicates. The procedure was as follows: 1) fixed the stems in the FAA solution (70% ethanol: glacial acetic acid: formaldehyde; 90: 5: 5, v/v) and embedded them in the frozen sectioning medium (OCT; Thermo Scientific, Waltham, MA); 2) cut the embedded stems into slices and put the slices on the slides; 3) stained the slides with phloroglucinol solution for 2 min; 4) soaked the slides in 50% (v/v) HCl; 5) put coverslips on the slides and wiped the slides with lens paper; 6) examined the slides with optical light microscope [47].

### Statistics analysis

All the data in the study were the mean and standard error of three biological replicates. Student’s t-test was used to identify significant differences between TLs and CLs. And the statistical significance was controlled at *p* < 0.05.

## Supplementary information

**Additional file 1:** **Table S1.** List of lignin- and cellulose-related genes in tobacco.

**Additional file 2: Table S2.** List of primer pairs.

**Additional file 3: Excel S1.** The FPKM information of differentially expressed NAC genes in the roots, stems and leaves of *Populus simonii×P. nigra. (XLS 53 kb)*

**Additional file 4: Figure S1.** Nuclear localization of NAC15 with DAPI staining. A, dark field for DAPI; B, dark field for GFP; C, bright field; D, overlay of DAPI and GFP. Scale bar = 20 μm.

**Additional file 5: Figure S2.** Working model of NAC family TFs and their downstream genes in wood formation.

## Data Availability

All data generated or analysed during this study are included in this published article and its supplementary information files.

## Abbreviations

*C4H*: Cinnamate 4-hydroxylase
*CAD*: Cinnamyl alcohol dehydrogenase
*CCOMT*: Caffeoyl-CoA O-methyltransferase
*CesA*: Cellulose synthase
CL: Control line containing empty vector
*CL*: Coumarate: coenzyme A ligase
ESTs: Expressed sequence tags
FPKM: Fragment per kilo bases per million reads
GFP: Green fluorescent protein
*NST*: NAC secondary wall thickening
*PAL*: Phe ammonia-lyase
PCD: Programmed cell death
*PtrWNDs*: Wood-associated NAC master switches from poplar
*SND*: Secondary wall-associated NAC domain
TF: Transcription factor
TL: Transgenic line overexpressing *NAC15* gen
*VND*: Vascular-related NAC domain
*WND*: Wood-associated NAC domain
WT: Wild type
