# Supplementary material for: Over-expression of poplar NAC15 gene enhances wood formation in transgenic tobacco
Source: BMC Plant Biol. 2020 Jan 8;20:12. doi: 10.1186/s12870-019-2191-2 (PMC6950812; doi:10.1186/s12870-019-2191-2)
Supplement: Supplementary file 1 — Additional file 1: Table S1. List of lignin- and cellulose-related genes in tobacco. [file 12870_2019_2191_MOESM1_ESM.docx]

Table S1 The information of the up-regulated genes

| Name | Gene Accession | Gene Definition | Pathways | References |
| --- | --- | --- | --- | --- |
| CesA1 | NM_001326276 | cellulose synthase A catalytic subunit 2 | Cellulose synthase | Wada Y., Miyamoto K., Kusano T., et al. (2004). Association between up-regulation of stress-responsive genes and hypomethylation of genomic DNA in tobacco plants. *Molecular Genetics and Genomics*, *271*(6), 658-666. |
| CesA7 | JQ735443 | *NtCesA7*-24-5 cellulose synthase (cesA) | Cellulose synthase | Goué N., Mortimer J.C., Nakano Y., et al. (2013). Secondary cell wall characterization in a BY-2 inductive system. Plant Cell, Tissue and Organ Culture (PCTOC), 115(2), 223-232. |
| CesA8 | JQ735445 | *NtCesA8*F8-4 cellulose synthase (cesA) | Cellulose synthase | Goué N., Mortimer J.C., Nakano Y., et al. (2013). Secondary cell wall characterization in a BY-2 inductive system. Plant Cell, Tissue and Organ Culture (PCTOC), 115(2), 223-232. |
| IRX8 | JQ735447 | *NtIRX8F528d* glycosyl transferase (IRX8) | Secondary cell wall biosynthesis | Goué N., Mortimer J.C., Nakano Y., et al. (2013). Secondary cell wall characterization in a BY-2 inductive system. Plant Cell, Tissue and Organ Culture (PCTOC), 115(2), 223-232. |
| IRX10 | JQ735451 | *NtIRX-F21* glycosyl transferase (IRX10) | Secondary cell wall biosynthesis | Goué N., Mortimer J.C., Nakano Y., et al. (2013). Secondary cell wall characterization in a BY-2 inductive system. Plant Cell, Tissue and Organ Culture (PCTOC), 115(2), 223-232. |
| PAL1 | AB008200 | palB gene for phenylalanine ammonia-lyase | Lignin biosynthesis | Taguchi G., Sharan M., Gonda K., et al. (1998). Effect of methyl jasmonate and elicitor on PAL gene expression in tobacco cultured cells. *Journal of plant biochemistry and biotechnology*, *7*(2), 79-84. |
| PAL4 | NM_001325544 | phenylalanine ammonia-lyase | Lignin biosynthesis | Pellegrini L., Rohfritsch O., Fritig B., et al. (1994). Phenylalanine ammonia-lyase in tobacco (molecular cloning and gene expression during the hypersensitive reaction to tobacco mosaic virus and the response to a fungal elicitor). *Plant Physiology,* *106*(3), 877-86. |
| CAD14/19 | NM_001325471 | cinnamyl alcohol dehydrogenase 1 | Lignin biosynthesis | Knight M.E., Halpin C., Schuch W.. (1992). Identification and characterisation of cdna clones encoding cinnamyl alcohol dehydrogenase from tobacco. *Plant Molecular Biology,* *19*(5), 793. |
| 4CL1 | NM_001325738 | 4-coumarate--CoA ligase 1 | Lignin biosynthesis | Lee D., and Douglas C.J. (1996). Two divergent members of a tobacco 4-coumarate: coenzyme a ligase (4cl) gene family. cdna structure, gene inheritance and expression, and properties of recombinant proteins. *Plant Physiology,* *112*(1), 193-205. |
| 4CL2 | NM_001325625 | 4-coumarate--CoA ligase 2 | Lignin biosynthesis | Lee D., and Douglas C.J. (1996). Two divergent members of a tobacco 4-coumarate:coenzyme a ligase (4cl) gene family. cdna structure, gene inheritance and expression, and properties of recombinant proteins. *Plant Physiology,* *112*(1), 193-205. |
| HCT | NM_001325623 | shikimate O-hydroxy cinnamoyl transferase | Lignin biosynthesis | Zago, & E. (2006). Nitric oxide- and hydrogen peroxide-responsive gene regulation during cell death induction in tobacco. *PLANT PHYSIOLOGY,* *141*(2), 404-411. |
| CCOMT | NM_001325400 | caffeoyl-CoA O-methyl transferase 1 | Lignin biosynthesis | Martz F., Maury S., Pinçon G., et al. (1998). cDNA cloning, substrate specificity and expression study of tobacco caffeoyl-CoA 3-O-methyltransferase, a lignin biosynthetic enzyme. Plant molecular biology, 36(3), 427-437. |
| C4H | AB236952 | C4H mRNA for trans-cinnamate 4-monooxygenase | Secondary cell wall biosynthesis | Gális I., Šimek P., Narisawa T., et al. (2006). A novel R2R3 MYB transcription factor NtMYBJS1 is a methyl jasmonate‐dependent regulator of phenylpropanoid‐conjugate biosynthesis in tobacco. The Plant Journal, 46(4), 573-592. |
