# Supplementary material for: Over-expression of poplar NAC15 gene enhances wood formation in transgenic tobacco
Source: BMC Plant Biol. 2020 Jan 8;20:12. doi: 10.1186/s12870-019-2191-2 (PMC6950812; doi:10.1186/s12870-019-2191-2)
Supplement: Supplementary file 2 — Additional file 2: Table S2. List of primer pairs. [file 12870_2019_2191_MOESM2_ESM.doc]

Table S2 The primer sequences

| Primer Name | Forward primers | Reverse primers |
| --- | --- | --- |
| NAC15-1 | GGTTGATCTTAATAAGCTTGAGC | GCATGATCCAATCGGATTTC |
| NAC15-2 | GCTCTAGAATGCCTGAGGATATGATGAATC | GACTAGTTACCGATAAGTGGCATAATGG |
| NAC15-3 | GCTCTAGAATTGATCTCTAGCTACTCTCC | GACTAGTTGGTGTGAATTGACTATGCC |
| NAC15-4 | ATTGATCTCTAGCTACTCTCC | TGGTGTGAATTGACTATGCC |
| Reference | GATCTTGCTGGTCGTGATCT | ACTTCCGGACATCTGAACCT |
| CesA1 | TATGGATACGGGACTGTTGCATGG | TACCACCACCTTTACTGCCTCCG |
| CesA7 | TGCTTCGTCCAGTTTCCTCAAAG | CTGGACCTTGTATTCCGTCTAGG |
| CesA8 | CCAAAGCTGCAGATGATGGAGAGT | TTGAGTGCATCAGAGAAACCAGCA |
| IRX8 | TGAGCATTCTACCAATTCCAATGC | CTGCACGAGTGATGAAGCAACAAC |
| IRX10 | CCCTGTTTACACCACTTGTGACCT | AGAAATGATCAGCTCCCTCTGTCC |
| PAL1 | ATTGGAGCTTTCGAAGATGAATTG | CTGTTCCAAGCTCCTTTCTCACAA |
| PAL4 | CTGCAATTGCCAACAGGATAAAGG | TGTCCATTGCACATTGCTGTGAAC |
| CAD14 | CTGTTGGCCATCCTCTTGAACCTT | CTTCCTGTGATGCTCTTTCTCCCG |
| CAD19 | TGATACTGTCCCTGTTGGCCATCC | TTCCAGTGATGCTCTTTCTCCCGA |
| 4CL1 | ATGGTTACACACTGGCGACATTGG | ACAGCAGCATCAGAAATGTTGGGA |
| 4CL2 | TGAGCTCGAAGCTCTCCTTCTCAA | CTTCAGTAATGGTGGATCCGTTGG |
| HCT | ACTCCTATTGCAGTCGCAGGTGAT | ACGTATGTGCACCACGAACAAGAG |
| CCOMT | CCTGAGCCCATGAAAGAGCTAAGA | CAATCTCCATTGTGTTCTTGGCAT |
| C4H | GAGAGCAAGATCCTGGTAAACGCT | AGCTCCTCCTACCAACACCAAATG |

Note: TCTAGA-Xbal I; ACTAGT-Spe I
