# Supplementary figures and images for: Over-expression of poplar NAC15 gene enhances wood formation in transgenic tobacco
Source: BMC Plant Biol. 2020 Jan 8;20:12. doi: 10.1186/s12870-019-2191-2 (PMC6950812; doi:10.1186/s12870-019-2191-2)

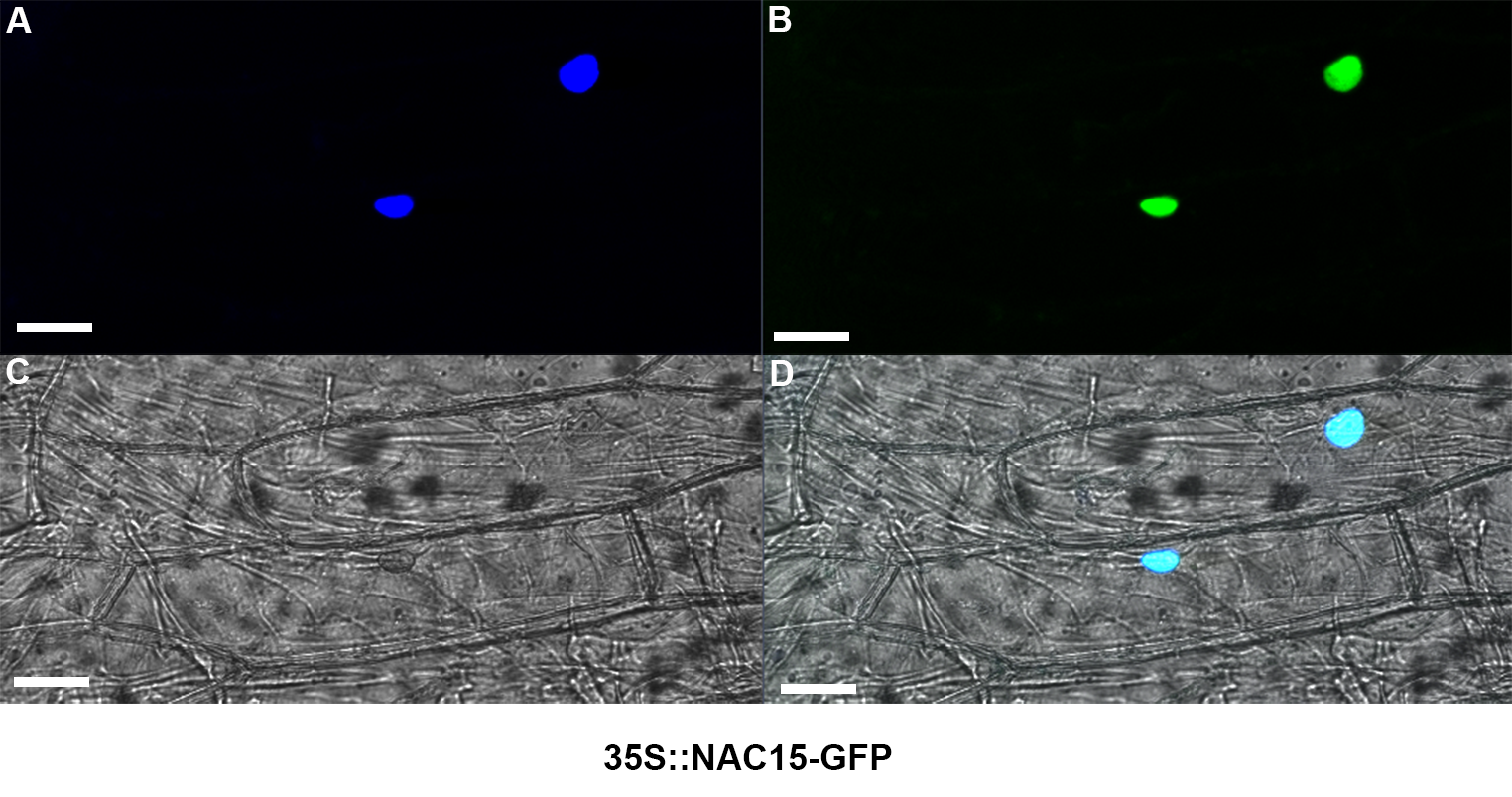

Supplement: Supplementary file 4 — Additional file 4: Figure S1. Nuclear localization of NAC15 with DAPI staining. A, dark field for DAPI; B, dark field for GFP; C, bright field; D, overlay of DAPI and GFP. Scale bar = 20 μm. [file 12870_2019_2191_MOESM4_ESM.tif]

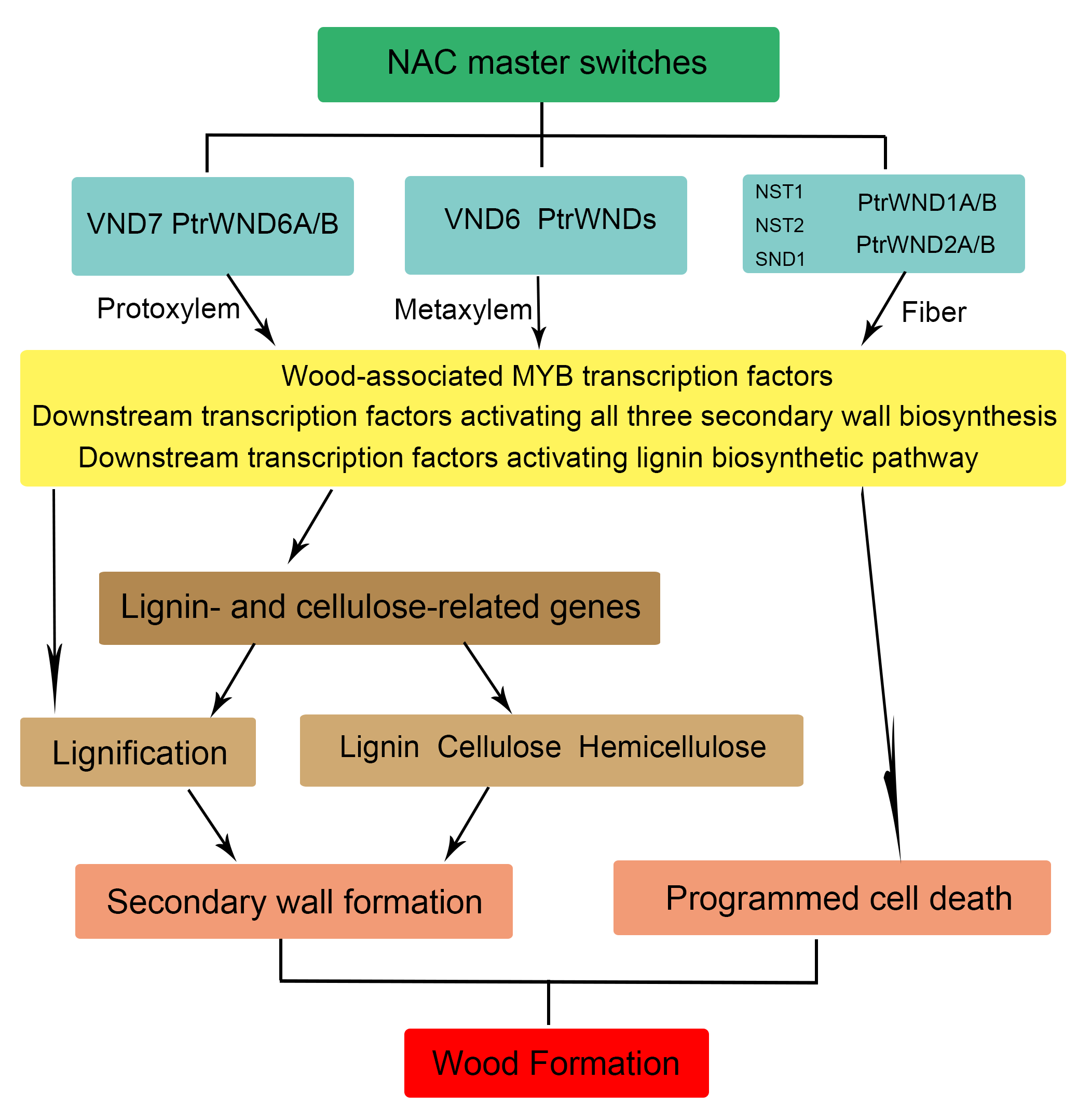

Supplement: Supplementary file 5 — Additional file 5: Figure S2. Working model of NAC family TFs and their downstream genes in wood formation. [file 12870_2019_2191_MOESM5_ESM.tif]
